# Characterization of *Vibrio cholerae* Hfq Provides Novel Insights into the Role of the Hfq C-Terminal Region

**DOI:** 10.1016/j.jmb.2012.03.028

**Published:** 2012-06-29

**Authors:** Helen A. Vincent, Charlotte A. Henderson, Timothy J. Ragan, Acely Garza-Garcia, Peter D. Cary, Darren M. Gowers, Marc Malfois, Paul C. Driscoll, Frank Sobott, Anastasia J. Callaghan

**Affiliations:** 1 Biophysics Laboratories, School of Biological Sciences, Institute of Biomedical and Biomolecular Sciences, University of Portsmouth, Portsmouth PO1 2DY, UK; 2 Division of Molecular Structure, MRC National Institute for Medical Research, The Ridgeway, Mill Hill, London NW7 1AA, UK; 3 Diamond Light Source Ltd., Harwell Science and Innovation Campus, Didcot OX11 0DE, UK; 4 Department of Biochemistry, University of Oxford, South Parks Road, Oxford OX1 3QU, UK

**Keywords:** sRNA, small RNA, VcHfq, *Vibrio cholerae* Hfq, EcHfq, *Escherichia coli* Hfq, NTD, N-terminal domain, CTR, C-terminal region, SAXS, small-angle X-ray scattering, AUC, analytical ultracentrifugation, EMSA, electrophoretic mobility shift assay, Qrr, quorum regulatory RNA, PDB, Protein Data Bank, PONDR, Predictors of Natural Disordered Regions, EDTA, ethylenediaminetetraacetic acid, sRNA, gene regulation, phylogenetic analysis, small-angle X-ray scattering, RNA binding

## Abstract

Hfq is a bacterial RNA binding protein that facilitates small RNA-mediated posttranscriptional gene regulation. In *Vibrio cholerae*, Hfq and four Hfq-dependent small RNAs are essential for the expression of virulence genes, but little is known about this mechanism at the molecular level. To better understand *V. cholerae* Hfq structure and mechanism, we characterized the protein, alongside *Escherichia coli* Hfq for comparison, using biochemical and biophysical techniques. The N-terminal domain (NTD) of the two proteins is highly conserved, but the C-terminal regions (CTRs) vary in both sequence and length. Small-angle X-ray scattering studies showed that both proteins adopt a star-shaped hexameric structure in which the conserved NTD adopts the expected Sm fold while the variable CTR is disordered and extends radially outwards from the folded core. Despite their structural similarity, SDS-PAGE stability assays and collision-induced dissociation mass spectrometry revealed that the *V. cholerae* hexamer is less stable than that of *E. coli*. We propose that this is due to minor differences between the intersubunit interface formed by the NTDs and the ability of the *E. coli* CTR to stabilize this interface. However, based on electrophoretic mobility shift assays, the divergent CTRs do appear to perform a common function with regard to RNA-binding specificity. Overall, the similarities and differences in the fundamental properties of *V. cholerae* and *E. coli* Hfq provide insight into their assembly and molecular mechanisms.

## Graphical Abstract

**Figure:**
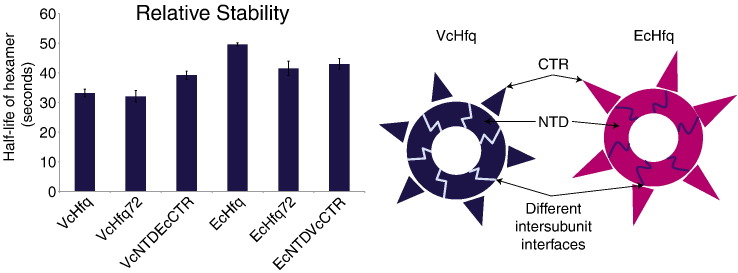

**Highlights:**

► This is the first study of *V. cholerae* Hfq, an RNA binding protein that regulates virulence genes. ► The NTD is homologous to *E. coli* Hfq, the CTR is not. ► Both *V. cholerae* and *E. coli* Hfq CTRs extend outwards from a hexameric NTD core. ► Both *V. cholerae* and *E. coli* Hfq CTRs enhance the RNA binding specificity of Hfq. ► Only the *E. coli* CTR stabilizes the Hfq hexamer.

## Introduction

Hfq is a bacterial RNA binding protein that is required for the facilitation of small RNA (sRNA)-mediated posttranscriptional gene regulation (reviewed in Ref. 1). sRNAs typically base pair to their mRNA target(s) to modulate translation and turnover of the mRNA. The most common outcome is repression of translation through binding of the sRNA to the ribosome-binding site of the mRNA, and this is often coupled to enhanced degradation of the mRNA. Alternatively, translation may be stimulated when sRNA binding remodels secondary structure in the mRNA that would otherwise prevent access to the ribosome-binding site.

There are at least 80 sRNAs in *Escherichia coli*, and other bacterial genomes may encode several hundred.^1^ For example, a high-throughput screen identified approximately 500 putative sRNAs in *Vibrio cholerae*.^2^ The predominant physiological role for sRNAs is in modulating stress responses to allow bacteria to adapt to environmental pressures, for example, transition to stationary phase, osmotic stress, and low iron concentrations (reviewed in Ref. 1). Often the primary mRNA target is a transcription factor that will propagate the signal to downstream mRNAs to bring about a global response.^1^ It is becoming more apparent that Hfq-dependent sRNA-mediated gene regulation is required for virulence phenotypes in pathogenic bacteria (reviewed in Ref. 3). This requirement is due to both the adaptive responses necessary for growth and fitness and also the expression of specific virulence factors and secretion systems being controlled by sRNAs.^3^

In *V. cholerae*, Hfq-dependent sRNAs ensure differential expression of genes required for virulence and biofilm formation in response to cell density.^4–9^ At the low cell densities present during the onset of infection, four sRNAs (the quorum regulatory RNAs or Qrrs 1–4) are synthesized.^7^ Hfq facilitates the pairing of the Qrrs with *hapR*^7,10^ and *vca0939*^8^ mRNAs (*hapR* encodes the transcription factor HapR, which is required for the repression of virulence genes,^7^ and *vca0939* is predicted to control the levels of the second messenger cyclic di-GMP^8^). This pairing results in the repression of *hapR* translation^7^ and the stimulation of *vca0939* translation,^8^ which, in turn, lead to the expression of virulence genes.^7–9^ At the high cell densities present at the late stages of infection, the Qrrs are no longer synthesized.^5^ In the absence of the sRNAs, *hapR* is translated,^7^ translation of *vca0939* is repressed,^8^ the genes required for virulence are repressed,^7–9^ and the bacterium is released from the host.

The mechanisms by which Hfq facilitates such sRNA-mediated gene regulation are not well understood (reviewed in Ref. 11). In an RNA-binding capacity, Hfq may increase the local concentrations of the sRNAs and mRNAs to stimulate base pairing or protect the RNA from ribonuclease activity. As an RNA chaperone, it could alter RNA structure to remove secondary structures that are inhibitory for pairing of the sRNA and mRNA or protect or expose ribonuclease cleavage sites. Hfq might also be involved in ribonucleoprotein complex formation, recruiting ribonucleases or ribosomes to the mRNA. The situation is further complicated because the precise role or combination of roles that Hfq performs may depend upon the identity of the sRNA and/or mRNA. Understanding the mechanism of Hfq at the molecular level will be essential for fully understanding sRNA-mediated gene regulation. As a first step to understanding the role that Hfq plays in the pathogenicity of *V. cholerae*, the protein has been characterized using biochemical and biophysical techniques. Comparison with Hfq from *E. coli* reveals both similarities and differences between the two proteins, which provide novel insights into the function of the divergent C-terminal region (CTR) of Hfq proteins.

## Results

### Sequence comparison of *V. cholerae* Hfq and *E. coli* Hfq

Extensive bioinformatics analyses have shown that Hfq is highly conserved and widely distributed throughout bacteria.^12–15^ The N-terminal domain (NTD; residues 1–72) is most similar between species, while the CTR varies significantly in both its length and amino acid sequence.^14,15^ Phylogentically, Hfq can be separated into three clades: alphaproteobacteria, betaproteobacteria/gammaproteobacteria, and low GC Gram-positive bacteria.^12^ Since the gammaproteobacteria is a taxonomic class rich in pathogenic genera, including *V. cholerae* and *E. coli*, this work focuses upon this class.

**Fig. 1:**
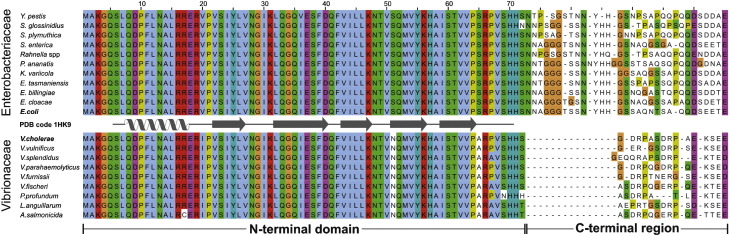
Multiple sequence alignment of representative members of the gammaproteobacteria families Enterobacteriaceae and Vibrionaceae. Only nonredundant sequences (<   95% amino acid identity) are shown. The boundaries for the NTD and CTR are indicated. The depicted regular secondary-structure elements correspond to the crystal structure of *E. coli* Hfq (EcHfq) (PDB ID: 1HK9)^14^ with β-strands as arrows and the α-helix as a spiral. Positions are colored according to the Clustal color scheme: glycines are orange and prolines are yellow; other positions are colored according to conservation of chemical properties: hydrophobic in blue, aromatic in cyan, polar negative in purple, polar positive in red, and polar neutral in green. An alignment including other gammaproteobacteria families is shown in Fig. S2.

Phylogenetic analyses were performed by both maximum likelihood and Bayesian approaches for gammaproteobacteria Hfqs. The consensus trees presented in Fig. S1 have similar topology and show that Hfq proteins cluster according to family; for example, *V. cholerae* Hfq (VcHfq) clusters with Hfqs from other *Vibrio* species. Multiple sequence alignments for gammaproteobacteria Hfq protein sequences are shown in Fig. 1 and Fig. S2. As expected, the sequence conservation is very high in the NTD, with an identity at the amino acid sequence level of 80% between the most divergent sequences in the data set. Of 72 positions, 69 are identical between the VcHfq and the *E. coli* Hfq (EcHfq) NTDs. In contrast, the length and composition of the CTR vary greatly within the data set, but less within each taxonomic family; for example, among the *E. coli* family Enterobacteriaceae, the CTR is between 27 and 31 residues long with a sequence identity between 39% and 83%; while for the *V*. *cholerae* family Vibrionaceae, the CTR is between 13 and 16 residues long with a sequence identity between 35% and 97%. However, the sequence identity between the *V. cholerae* and *E. coli* CTRs is only 6%.

### Structural analyses of VcHfq

In the crystal structures of EcHfq, the NTD forms a hexamer arrayed in the torus shape characteristic of Sm protein domains.^14,16,17^ The Sm fold consists of an N-terminal α-helix followed by a five-stranded antiparallel β-sheet; in the quaternary structure, a 30-stranded interchain antiparallel β-sheet is formed, mediated by β-strands 4 and 5 of adjacent monomers.^14,16,17^ Recent small-angle X-ray scattering (SAXS) studies have shown that full-length EcHfq hexamer adopts a six-pointed star conformation, indicating that the CTR extends radially outwards from the folded NTD core.^18^

Based on the level of homology between the VcHfq and EcHfq protein sequences, it is likely that the VcHfq NTD core folds into a similar compact hexameric structure to that of EcHfq. However, the expected conformation of the VcHfq CTR is less clear. Analytical ultracentrifugation (AUC), SAXS, homology modeling, secondary-structure prediction algorithms, and circular dichroism (CD) were all employed to probe the structure of full-length VcHfq in solution.

**Fig. 2:**
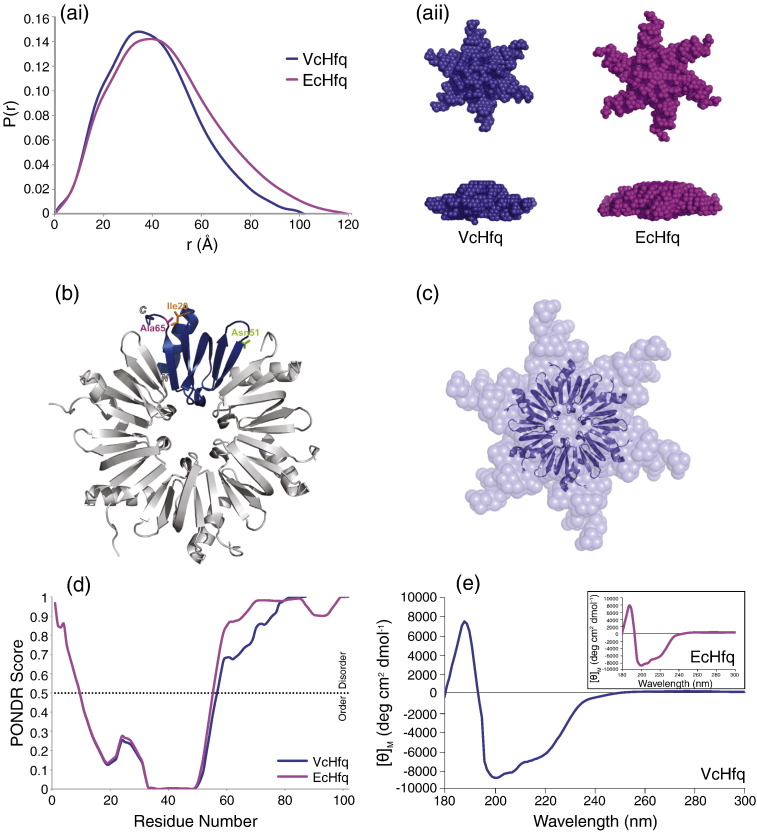
Structural studies of VcHfq and EcHfq. (a) SAXS. (i) Pair distance distribution function, *P*(*r*), plots for VcHfq (blue) and EcHfq (magenta) calculated by an indirect Fourier transformation of the SAXS data using GNOM.^19^ (ii) Surface representations of *ab initio* models for VcHfq (left; blue) and EcHfq (right; magenta) calculated using DAMMIF^20^ and visualized with PyMOL. Top and side views are shown for each protein. (b) Homology model of the VcHfq NTD. A ribbon representation of a homology model of the VcHfq NTD hexamer visualized with PyMOL. One monomer has been colored blue and the three positions that differ from the EcHfq NTD are highlighted on this monomer. One hundred models were calculated using MODELLER 9v8^21^ with the structure of the EcHfq hexamer (PDB ID: 1HK9)^14^ as the template. The model shown is the one with the lowest discrete optimized protein energy potential. (c) Superposition of the VcHfq NTD homology model onto the *ab initio* model of VcHfq generated from the SAXS data. The structures were superimposed using SUPCOMB^22^ and visualized with PyMOL. (d) PONDR predictions for VcHfq and EcHfq. Potentially disordered regions of VcHfq (blue) and EcHfq (magenta) were identified using the PONDR VL-XT algorithm.^23–25^ The output from the predictor is a value between 0 and 1 with values of 0.5 and above indicating that a given amino acid is disordered. (e) CD spectra for VcHfq and EcHfq. The CD spectrum for VcHfq from 180 nm to 300 nm is shown in the main panel and the CD spectrum for EcHfq, over the same wavelength range, is shown as an inset.

The elution volume of VcHfq from the size-exclusion column during the final purification step is consistent with the *V. cholerae* protein existing as a hexamer, and AUC (Fig. S3) confirmed that both VcHfq and EcHfq are hexameric in solution. SAXS was used to determine the molecular envelopes of VcHfq and EcHfq and the pair distance distribution, *P*(*r*), plots are shown for both proteins in Fig. 2a,i. Molecular masses of 55 kDa and 61 kDa for VcHfq and EcHfq, respectively, were estimated from the SAXS profiles and are consistent with both proteins forming hexamers.

The radius of gyration (*R*_g_), from Guinier analysis, is 31 Å for VcHfq and 36 Å for EcHfq, and the maximum particle dimension (*D*_max_) is 103 Å for VcHfq and 120 Å for EcHfq. The *R*_g_ and *D*_max_ for EcHfq are similar to those reported by Beich-Frandsen *et al.*^18^ and comparison of the *R*_g_ and *D*_max_ values suggests that the *V. cholerae* protein is slightly smaller than the *E. coli* protein, as might be expected from its shorter CTR. Representative *ab initio* models for each protein are shown in Fig. 2a,ii and are consistent with those recently presented for EcHfq.^18^ The models show both VcHfq and EcHfq in a six-pointed star conformation with the center of the star 65–70 Å in diameter and 35–40 Å thick and the points extending outwards for ∼ 40 Å for VcHfq and ∼ 50 Å for EcHfq. Since the NTDs of VcHfq and EcHfq differ by only three amino acids (Ile20, Asn51, and Ala65 in VcHfq are Val, Ser, and Ser in EcHfq, respectively), the structure of EcHfq [residues 6–70, Protein Data Bank (PDB) ID: 1HK9] was used as a template to build a homology model of the NTD of VcHfq (Fig. 2b). This homology model can be superimposed onto the body of the star in the model produced from the SAXS data (Fig. 2c), implying that, as for EcHfq,^18^ the CTR creates the points of the star.

AUC, secondary-structure prediction algorithms, and CD all support the CTR of both VcHfq and EcHfq adopting an extended conformation. Frictional ratios calculated from AUC sedimentation velocity experiments are typically between 1.2 and 1.3 for globular proteins, with higher ratios suggesting that a protein is asymmetric and/or partially unfolded.^26^ For VcHfq and EcHfq, the frictional ratios were 1.95 and 1.81, respectively, consistent with their CTRs being intrinsically disordered. The output from the Predictors of Natural Disordered Regions (PONDR) VL-XT algorithm^23–25^ is shown in Fig. 2d for both VcHfq and EcHfq and strongly predicts that the NTD will be ordered and the CTR disordered in each case. Finally, the secondary-structure content of VcHfq and EcHfq (Table 1) was determined from the CD spectra shown in Fig. 2e. The two CD spectra are extremely similar and characteristic of proteins containing both α and β secondary structures. The number of residues in α or β structures is similar for both VcHfq and EcHfq and is consistent with the secondary-structure elements found within the NTD^14,17^ (Table 1). An extended conformation for the CTRs would account for the large number of disordered residues present in both proteins (Table 1) with the relative length of the CTRs explaining the fact that VcHfq contains fewer disordered residues than EcHfq (Table 1).

Taken together, these results suggest that the structure of VcHfq is very similar to that of EcHfq. Both proteins are hexameric, have an NTD that adopts the Sm fold, and have a disordered CTR that extends radially away from the NTD core resulting in a six-pointed star-shaped conformation. The VcHfq star is slightly smaller than that of EcHfq due to the shorter length of its CTR.

### Relative stability of the VcHfq and EcHfq hexamers

**Fig. 3:**
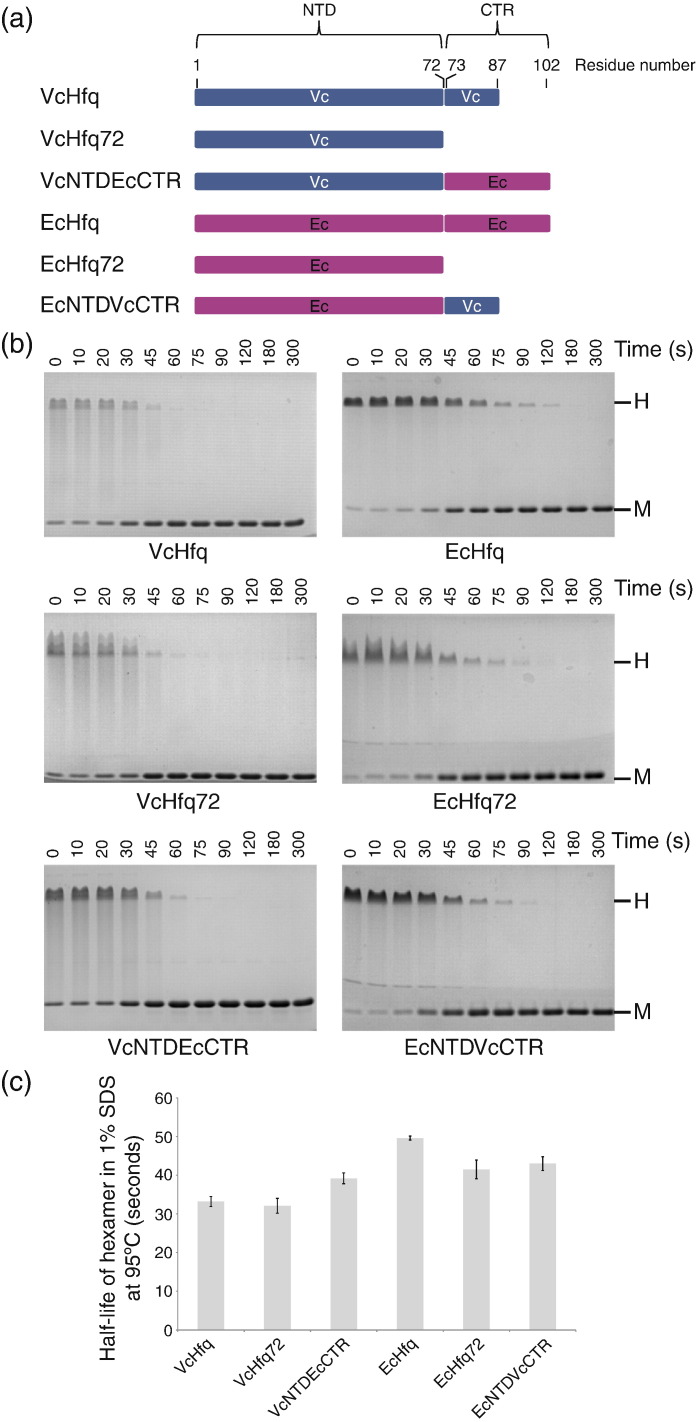
Relative stability of Hfq hexamers. (a) Schematic representation of the Hfq constructs used in this study. (b) Representative SDS-PAGE gels for VcHfq, VcHfq72, VcNTDEcCTR, EcHfq, EcHfq72, and EcNTDVcCTR heated in 1% SDS at 95 °C for 0, 10, 20, 30, 45, 60, 75, 90, 120, 180, or 300 s. Protein bands corresponding to protein hexamers and monomers are labeled on the right with the letter H or letter M, respectively. (c) Half-lives of the VcHfq, VcHfq72, VcNTDEcCTR, EcHfq, EcHfq72, and EcNTDVcCTR hexamers in 1% SDS at 95 °C. Error bars represent the standard error of the mean for three replicates.

Despite their similar structures, the VcHfq hexamer is less stable than the EcHfq hexamer. On a standard denaturing SDS-PAGE gel, when the protein has been heated at 95 °C for 60 s in the presence of 1% SDS prior to loading, VcHfq migrates entirely as a monomer. In contrast, a significant fraction of EcHfq continues to migrate as a hexamer, resistant to the SDS (compare the 60-s time point for VcHfq and EcHfq in Fig. 3b).

A similar difference in the relative stability of the two proteins was observed by non-denaturing mass spectrometry. Using identical experimental parameters, which were selected to preserve intact non-covalent interactions in the mass spectrometer, both VcHfq and EcHfq exist as stable hexamers (Fig. S4a). The 16+ charge states for each protein hexamer were chosen for fragmentation by collision-induced dissociation prior to the ion mobility cell. In collision-induced dissociation experiments, as collision energy is increased, non-covalent complexes dissociate into monomers and remaining complex.^27^ For VcHfq and EcHfq, the disappearance of hexamer and the appearance of monomer were monitored. Tandem mass spectrometry product-ion spectra are shown in Fig. S4b for both VcHfq and EcHfq at three different trap collision energies: 45 V, 55 V, and 120 V. As can be seen from the relative heights of the hexamer and monomer peaks at the different collision energies, the VcHfq hexamer fragments at a lower collision energy than the EcHfq hexamer.

Given that the NTDs of VcHfq and EcHfq differ by only three amino acids whereas their CTRs differ significantly in amino acid composition and length, it was expected that the observed stability difference would originate from the CTRs. VcHfq72 and EcHfq72, which are truncations containing only the NTD of VcHfq and EcHfq, respectively, and VcNTDEcCTR and EcNTDVcCTR, in which the CTR has been swapped between the two proteins, were constructed to investigate this hypothesis (a schematic representation of these proteins is shown in Fig. 3a). The relative stability of the proteins was then assessed by determining the half-life of the hexamers in 1% SDS at 95 °C using the SDS-PAGE assay. Representative SDS-PAGE gels are shown in Fig. 3b for VcHfq, EcHfq, VcHfq72, EcHfq72, VcNTDEcCTR, and EcNTDVcCTR and the corresponding half-lives of the hexamers are presented in Fig. 3c and Table 2.

The CTR of EcHfq has been reported to stabilize the hexameric quaternary structure of the protein.^15^ Consistent with this, the half-life of the EcHfq72 hexamer in 1% SDS at 95 °C is 8 s shorter than that of the full-length protein (41.5 s compared to 49.6 s; Table 2). Surprisingly, the half-life of the VcHfq72 hexamer is only 32.1 s (Table 2), indicating that, despite the high sequence identity, the VcHfq NTD is intrinsically less stable than the EcHfq NTD. In contrast to the *E. coli* protein, the half-life of full-length VcHfq is similar to that of VcHfq72 (Table 2), suggesting that the VcHfq CTR does not stabilize the hexamer. Different roles for the VcHfq and EcHfq CTRs with respect to the stability of the hexameric structure are supported by the CTR exchange experiments. Substitution of the VcHfq CTR for that of EcHfq increases the stability of VcHfq (the half-life of VcNTDEcCTR is 39.2 s in 1% SDS at 95 °C compared to 33.2 s for VcHfq; Table 2). However, exchange of the EcHfq CTR for that of VcHfq reduces the stability of EcHfq to that of the EcHfq NTD alone (half-life: 43 s). Therefore, the stability difference between the VcHfq and EcHfq hexamers appears to be due to both higher intrinsic stability of the EcHfq NTD and a stabilizing contribution from the EcHfq CTR.

### Hfq binding to Qrr1 sRNA

**Fig. 4:**
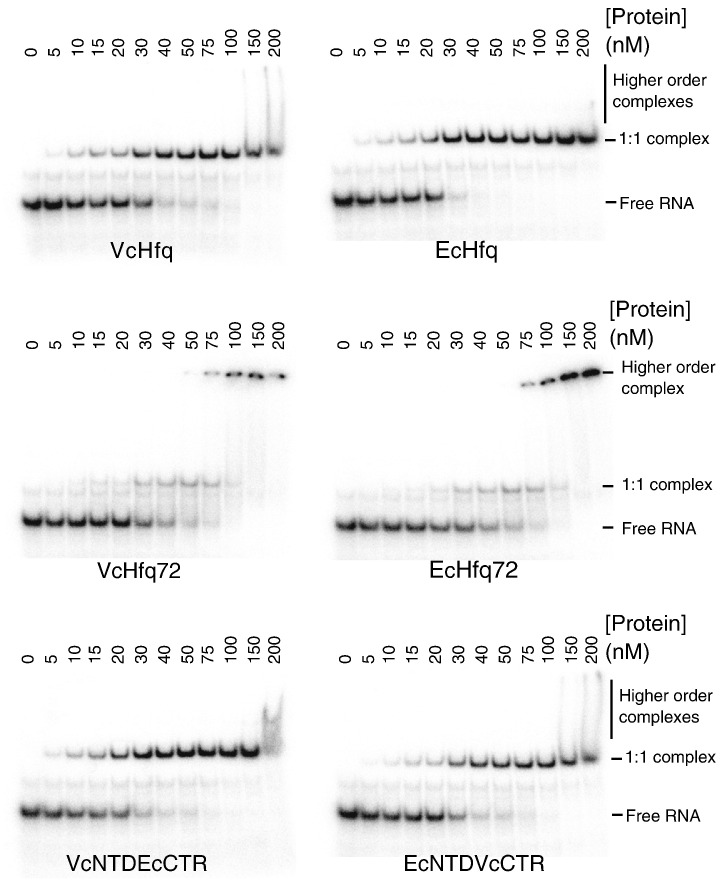
Hfq binding to Qrr1 sRNA. Representative EMSAs for VcHfq, VcHfq72, VcNTDEcCTR, EcHfq, EcHfq72, and EcNTDVcCTR binding to Qrr1 sRNA. ^32^P-labelled Qrr1 was at 5 nM and the concentrations of hexameric protein are specified above the lanes. The mobilities of free Qrr1 RNA, the 1:1 Qrr1:Hfq complex, and higher-order complexes are indicated on the right.

VcHfq is known to facilitate the pairing of the Qrr sRNAs to *hapR*^7,10^ and *vca0939*^8^ mRNAs and so the ability of VcHfq to bind to Qrr1 sRNA was investigated using an electrophoretic mobility shift assay (EMSA). The representative EMSA presented in Fig. 4 shows that a discrete 1:1 VcHfq:Qrr1 complex forms upon addition of increasing amounts of VcHfq to Qrr1, with continued addition of VcHfq leading to the formation of a less defined higher-order complex with multiple VcHfq molecules bound to a single RNA molecule. Dissociation constants (*K*_d_) were determined to be ∼ 30 nM for VcHfq binding to Qrr1 in a 1:1 complex and ∼ 200 nM for VcHfq binding to form a higher-order complex (Table 3), suggesting that there is one tight binding site and a second, lower-affinity binding site for VcHfq on Qrr1.

To explore the binding of Hfq to Qrr1 further, we also performed EMSAs with EcHfq, VcHfq72, EcHfq72, VcNTDEcCTR, and EcNTDVcCTR (Fig. 4) and determined the *K*_d_ values for the resulting complexes (Table 3). Full-length EcHfq and the CTR-swap constructs (VcNTDEcCTR and EcNTDVcCTR) bound Qrr1 in a similar manner to full-length VcHfq. All three Hfqs bound Qrr1 with a *K*_d_ of 18–28 nM to form a 1:1 complex and then with lower affinity (150–420 nM) to form a poorly defined higher-order complex. In contrast, VcHfq72 and EcHfq72, which lack the CTR, bound to Qrr1 to form two discrete complexes (presumably 1:1 and 1:2 Qrr1:Hfq complexes), and the binding affinity of the Hfq to form each complex was similar (∼ 40 nM in each case). This suggests that in the absence of the CTR, the affinity for the first binding site is slightly reduced, whereas the affinity for the second site is substantially increased. Therefore, the CTR appears to affect the binding of both VcHfq and EcHfq to Qrr1.

## Discussion

The sequence identity for VcHfq and EcHfq is 96% for their NTDs but just 6% for their CTRs. The contrast between the sequence stasis of the NTD and the high degree of sequence variability of the CTR suggests that these two regions are under very different evolutionary selection processes. While the NTD is under strong purifying selection to preserve its function, the CTR seems to be under positive selection, a behavior that is often attributed to functional diversification. This suggests that the CTR is somehow able to modulate the biophysical and binding properties of the respective NTD, without compromising its function.

Both VcHfq and EcHfq adopt similar hexameric structures in solution (Fig. 2). The NTDs adopt the Sm fold (Fig. 2b and e) observed in the crystal structures of EcHfq^14,16,17^ and Hfq from other species, e.g., *Staphylococcus aureus*^13^ and *Pseudomonas aeruginosa*,^28^ while the CTRs are disordered (Fig. 2d and e) and extend away from the folded NTD to create a six-pointed star shape (Fig. 2a,ii). Despite their structural homology, the VcHfq hexamer is less stable than that of EcHfq due to contributions to stability from both the EcHfq NTD and CTR (Fig. 3 and Table 2).

Three amino acids differ between the VcHfq NTD (Ile20, Asn51, and Ala65) and the EcHfq NTD (Val20, Ser51, and Ser65). In the EcHfq crystal structures, Val20 and Ser65 are in close contact but do not form part of the interface between subunits.^14,17^ The double substitution for Ile and Ala at these positions in VcHfq would be unexpected to have any significant effects on the overall properties of the hexamer (see Fig. 2b for the position of Ile20 and Ala65 in the homology model of the VcHfq NTD). Ser51 in EcHfq is located at the beginning of β-strand 4 at the intermonomer interface,^14,17^ and although the substitution for Asn in VcHfq (Fig. 2b) is fairly conservative, it could alter the intersubunit interface within the NTD and explain the lower intrinsic stability of the VcHfq NTD relative to the EcHfq NTD.

Removal of part of the EcHfq CTR results in a conformational change at the intersubunit interface,^15^ implying that the CTR forms part of this interface. In support of this, the residues at the C-terminal end of the NTD in the crystal structure of the EcHfq NTD fold back against the protein core, in a groove created by the N-terminal α-helices,^14^ and SAXS studies have suggested that the first part of the EcHfq CTR packs against the NTD.^18^ Involvement of the CTR in the intersubunit interface could account for the additional stability of the full-length EcHfq protein relative to the NTD. The CTR-swap experiment (Fig. 3) also indicates that the EcHfq CTR can interact with and stabilize the VcHfq NTD hexamer. In contrast, the inability of the VcHfq CTR to stabilize either the VcHfq NTD or the EcHfq NTD implies that the VcHfq CTR does not form part of the Hfq intersubunit interface.

Full-length VcHfq and EcHfq appear to bind Qrr1 sRNA with similar affinity and specificity. Both proteins bind tightly to Qrr1, with *K*_d_ values in the low nanomolar range, to form a 1:1 complex. Higher-order complexes are formed at significantly higher concentrations of Hfq (Fig. 4) and are presumably the result of a second Hfq molecule binding to another, weaker affinity site, on the RNA. However, the possibility that the second Hfq molecule binds to the first Hfq molecule, to similarly form a Qrr1:Hfq complex with 1:2 stoichiometry, cannot be excluded.

The VcNTDEcCTR and EcNTDVcCTR proteins, in which the CTRs have been exchanged between VcHfq and EcHfq, bind Qrr1 in a manner similar to that of the wild-type proteins (Fig. 4). In contrast, VcHfq72 and EcHfq72, which contain the NTD alone, appear to bind Qrr1 with comparable affinity but reduced specificity. Both truncated proteins can form complexes with apparent 1:1 and 1:2 Qrr1:Hfq stoichiometry, and the affinity for both sites is in the low nanomolar range. The observation that the binding affinity is largely unaffected by the removal of the CTR explains reports that the removal of the EcHfq CTR does not affect the ability of EcHfq to bind RNA.^15^ Overall, the similar RNA-binding results obtained for both the VcHfq and EcHfq constructs suggest a common function for all Hfq CTRs in maintaining binding stoichiometry and specificity rather than a species-specific role.

It was originally reported that Hfq prefers to bind to poly(A) and single-stranded AU-rich regions of RNA.^29,30^ It is now widely accepted that the Hfq NTD presents two binding faces: the proximal face with specificity for AU-rich RNA and the distal face with specificity for poly(A) and A–R–N triplets (where R is a purine and N is any nucleotide).^14,16,31^ Since most sRNAs bind to the proximal face of Hfq,^31,32^ it is likely that both VcHfq and EcHfq bind an AU-rich region of Qrr1 sRNA with this face. There are also indications that Hfq can recognize secondary structure. Studies have shown that Hfq prefers single-stranded regions that are adjacent to stem loops^31,33–35^ and Hfq can bind tRNA, implying an ability to bind structured RNA in the absence of a single-stranded region.^36^ If the proximal face of the conserved NTD core binds a single-stranded AU-rich region, the CTR, which extends from the proximal face,^14,17^ might detect other binding determinants, such as stem loops, in the RNA. In this manner, the CTR may assist in directing the RNA to the correct face of Hfq. In the absence of the CTR, only the sequence of the single-stranded region can be used as a binding determinant, resulting in lower specificity but only slightly reduced affinity. The effect that reduced binding specificity has on the formation of the correct Hfq:RNA complex required for riboregulation may be RNA dependent, which could explain the conflicting reports that the CTR is^37^ or is not^38^ required for Hfq function.

**Fig. 5:**
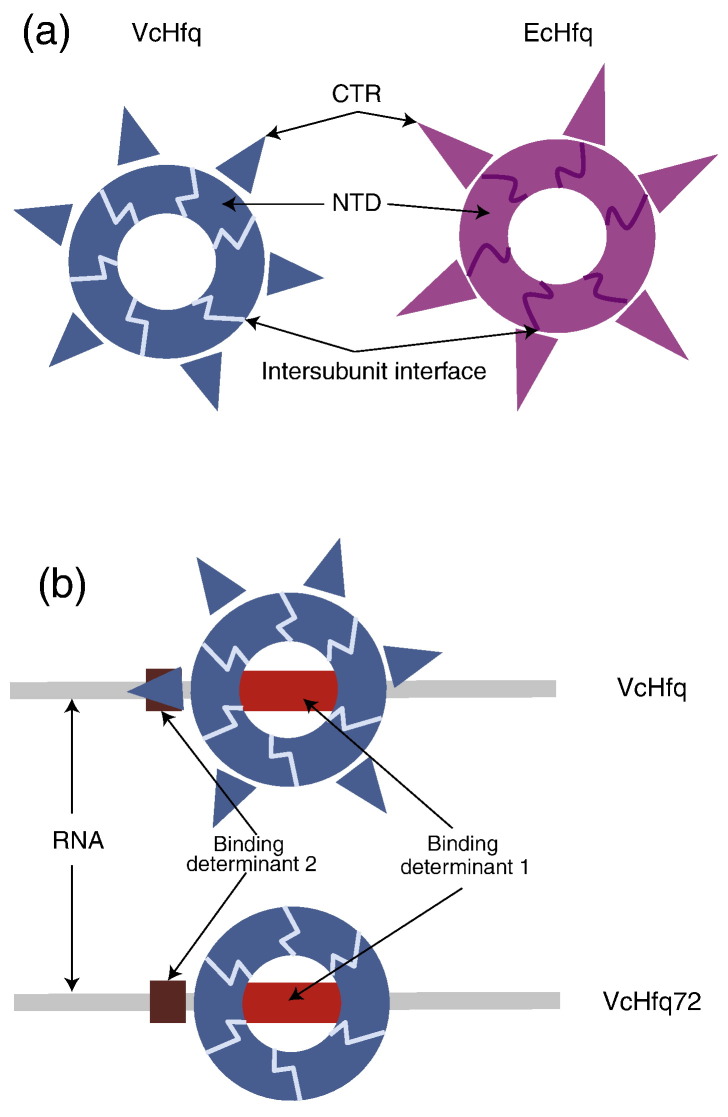
Model of VcHfq properties. (a) Model explaining the lower stability of the VcHfq hexamer relative to EcHfq. VcHfq and EcHfq NTDs are shown as blue and magenta rings, respectively, with the intersubunit interface indicated by a sharp zigzag line for VcHfq and a curvy zigzag line for EcHfq. The CTRs are represented by triangles that do not interact with the intersubunit interface of the VcHfq NTD but do contribute to the intersubunit interface of the EcHfq NTD. (b) Model explaining the contribution of the CTR to VcHfq binding. The full-length VcHfq protein (top) utilizes multiple binding determinants (bright and dark red boxes) to bind at a specific site on the RNA (gray). VcHfq72, consisting of only the NTD, can only bind to a single binding determinant (bright red box) resulting in reduced binding specificity.

In summary, the intersubunit interface appears to differ between VcHfq and EcHfq, resulting in lower stability of the VcHfq hexamer. As is shown schematically in Fig. 5a, the interface created by the NTDs is slightly different in VcHfq compared to EcHfq, despite the high degree of conservation. Furthermore, the CTR forms part of, and stabilizes, the intersubunit interface in EcHfq, but does not do so in VcHfq. Figure 5b explains the role of the CTR in binding site recognition. Full-length Hfq binds a single-stranded AU-rich region with its NTD while ‘sensing’ other binding determinants, such as stem loops, with its CTR. In the absence of the CTR, only AU-rich regions can be detected, resulting in reduced specificity.

It remains to be determined how the observed differences in the intersubunit interface between the *V. cholerae* and *E. coli* Hfq hexamers influence the mechanisms of Hfq-mediated posttranscriptional gene regulation in these and other organisms. Similarly, understanding how, despite differences in sequence and length, the CTR is able to perform a common function with regard to RNA binding in both organisms must be addressed in the future. Continuing to identify and explain these subtle variations between species will be essential when elucidating mechanisms at the molecular level.

## Materials and Methods

### Multiple sequence alignments

DNA multiple sequence alignments were calculated with MAFFT-G-INS-i^39^ and sequence manipulations were done using either Jalview^40^ or ClustalX.^41^

### Construction of Hfq expression plasmids

*V. cholerae hfq* was PCR-amplified from pANT7_cGST (PlasmID repository, Harvard Institute of Proteomics, Harvard Medical School, Massachusetts, USA) using the VcHfq primers (see Table S2 for sequences). *E. coli hfq* was a PCR-amplified from pEH-10(*hfq*) purified from the BL21(DE3), pEH-10(*hfq*) expression strain obtained from I. Moll (Max F. Perutz Laboratories, University of Vienna, Austria) using the EcHfq primers (Table S2). PCR products were cloned into pCR-Blunt II-TOPO (Invitrogen) using the Zero Blunt TOPO PCR Cloning Kit (Invitrogen) and each *hfq* was subcloned into the NdeI and XhoI sites of pET28b (Novagen) to generate pET28[VcHfq] and pET28[EcHfq] for expression of full-length VcHfq and EcHfq, respectively. pET28[VcHfq72] and pET28[EcHfq72] for expression of the NTD alone of VcHfq and EcHfq were generated by inserting a stop codon into pET28[VcHfq] and pET28[EcHfq] after the 72nd codon of Hfq using standard site-directed mutagenesis protocols and VcHfq72 primers (Table S2) for VcHfq and primers EcHfq72 (Table S2) for EcHfq. DNA sequences corresponding to the CTR of EcHfq and the CTR of VcHfq were generated by gene synthesis from overlapping primers^43^ using primers VcNTDEcCTR and EcNTDVcCTR (Table S2), respectively. The gene synthesis products were used as mega-primers for site-directed mutagenesis to insert the EcCTR after the 72nd codon of VcHfq in pET28[VcHfq] and the VcCTR after the 72nd codon of EcHfq in pET28[EcHfq]. Constructs were verified by sequencing.

### Expression of recombinant Hfq proteins

BL21(DE3), pET28[VcHfq]; BL21(DE3), pET28[VcHfq72]; BL21(DE3), pET28[VcNTDEcCTR]; BL21(DE3), pET28[EcHfq72]; and BL21(DE3), pET28[EcNTDVcCTR] were grown in LB supplemented with 50 μg/ml kanamycin at 37 °C until the absorbance at 600 nm (*A*_600_) reached 0.6. BL21(DE3), pEH-10(*hfq*) was grown in LB supplemented with 100 μg/ml ampicillin at 37 °C until the *A*_600_ reached 0.6. Expression was induced with 1 mM IPTG, and cells were incubated for a further 3 h at 37 °C. Cells were harvested by centrifugation at 5000*g*  for 20 min at 4 °C and the cell pellet was stored frozen at − 80 °C.

### Purification of recombinant Hfq proteins

Cell pellets from induced BL21(DE3), pET28[VcHfq]; BL21(DE3), pET28[VcHfq72]; BL21(DE3), pET28[VcNTDEcCTR]; BL21(DE3), pET28[EcHfq72]; and BL21(DE3), pET28[EcNTDVcCTR] were thawed on ice and resuspended in 20 mM Tris, pH 7.4, 500 mM NaCl, 10 mM MgCl_2_, 1 U/ml DNase I (Promega), and 0.5 mg/ml lysozyme (Sigma).  RNase A (0.1 mg/ml; Sigma) was added to the preparations of VcHfq72 and EcHfq72. Cells were disrupted by sonication, and the lysate was clarified by centrifugation at 40,000*g* for 30 min at 4 °C. After addition of imidazole to 20 mM, the supernatants were loaded onto a HisTrap HP column (GE Healthcare) equilibrated in 20 mM Tris, pH 7.4, 500 mM NaCl, and 20 mM imidazole at 4 °C using an ÄKTAxpress (GE Healthcare). Proteins were eluted with a linear gradient to 1 M imidazole. Hfq-containing fractions were buffer exchanged into 20 mM Tris, pH 8, 500 mM NaCl, 2.5 mM CaCl_2_, and 10% glycerol using a PD10 column (GE Healthcare) and incubated with 0.5 U thrombin (Sigma) at room temperature for 16 h. Imidazole was added to the VcHfq preparation to a final concentration of 20 mM before loading onto a HisTrap HP column equilibrated in 20 mM Tris pH 7.4, 500 mM NaCl, and 20 mM imidazole at 4 °C using an ÄKTAxpress. Protein was eluted with a linear gradient to 1 M imidazole. VcHfq-containing fractions and the VcNTDEcCTR and EcNTDVcCTR preparations were buffer exchanged into 20 mM Tris, pH 8, 1 M NaCl, 1 M (NH_4_)_2_SO_4_, and 0.5 mM ethylenediaminetetraacetic acid (EDTA) using a PD10 column and loaded onto a HiTrap Butyl-S FF column (GE Healthcare) equilibrated in 20 mM Tris, pH 8, 1 M NaCl, 1 M (NH_4_)_2_SO_4_, and 0.5 mM EDTA at 4 °C using an ÄKTAxpress. Proteins were eluted with a linear gradient to 0 M (NH_4_)_2_SO_4_. EcNTDVcCTR-containing fractions and the VcHfq72 and EcHfq72 preparations were buffer exchanged into 20 mM Hepes, pH 7, 100 mM NaCl, and 0.5 mM EDTA and loaded onto a Mono S 4.6/100 PE column (GE Healthcare) equilibrated in 20 mM Hepes, pH 7, 100 mM NaCl, and 0.5 mM EDTA at 4 °C using an ÄKTAxpress. Proteins were eluted in a linear gradient to 1 M NaCl. VcNTDEcCTR-containing fractions were buffer exchanged into 20 mM 4-morpholineethanesulfonic acid, pH 6, 100 mM NaCl, and 0.5 mM EDTA and loaded onto a Mono S 4.6/100 PE column equilibrated in 20 mM 4-morpholineethanesulfonic acid, pH 6, 100 mM NaCl, and 0.5 mM EDTA at 4 °C using an ÄKTAxpress. Proteins were eluted in a linear gradient to 1 M NaCl. All preparations were buffer exchanged into 20 mM Tris, pH 8, 500 mM NaCl, 0.5 mM EDTA, and 10% glycerol using a PD10 column, concentrated to ∼ 10 mg/ml using a VivaSpin 2 centrifugal concentrator with a molecular mass cutoff of 10 kDa and loaded onto a Superdex 200 10/300 GL size-exclusion column (GE Healthcare) equilibrated in 20 mM Tris, pH 8, 500 mM NaCl, 0.5 mM EDTA, and 10% glycerol using an ÄKTApurifier (GE Healthcare). Hfq-containing fractions were pooled and concentrated to ∼ 5 mg/ml using a VivaSpin 2 centrifugal concentrator with a molecular mass cutoff of 10 kDa. VcHfq, VcNTDEcCTR, EcHfq72, and EcNTDVcCTR were stored frozen at − 80 °C and VcHfq72 was stored at 4 °C.

EcHfq was purified from BL21(DE3), pEH-10(*hfq*) essentially as described in Vassilieva *et al.*^43^ except that following the hydrophobic interaction column, the EcHfq-containing fractions were concentrated to ∼ 10 mg/ml using a VivaSpin 2 centrifugal concentrator with a molecular mass cutoff of 10 kDa and loaded onto a Superdex 200 10/300 GL size-exclusion column equilibrated in 20 mM Tris, pH 8, 500 mM NaCl, 0.5 mM EDTA, and 10% glycerol using an ÄKTApurifier. EcHfq-containing fractions were pooled and concentrated to ∼ 10 mg/ml using a VivaSpin 2 centrifugal concentrator with a molecular mass cutoff of 10 kDa and stored frozen at − 80 °C.

### Small-angle X-ray scattering

SAXS data were collected using standard procedures at the bioSAXS beamline ID14-3^44^ at the European Synchrotron Radiation Facility (Grenoble, France) at a wavelength of 0.931 Å and a camera length of 2.42 m. The detector used was a Pilatus 1 M (Dectris), and the range of momentum transfer covered was 0.005 Å^− 1^ < *q* > 0.5 Å^− 1^. Data were collected at 25 °C for three concentrations of both VcHfq (9.51, 4.67, and 0.85 mg/ml in 10 mM Tris, pH 8, and 500 mM NaCl) and EcHfq (9.1, 4.25, and 0.85 mg/ml in 10 mM Tris, pH 8, and 500 mM NaCl) in order to be able to correct for interparticle effects. The data were normalized to the intensity of the incident beam and the scattering of the buffer was subtracted in PRIMUS.^45^ The data were collected in 10 successive  10-s frames with the sample continuously under flow to check for radiation damage and aggregation during the SAXS experiment. For all SAXS data, the radius of gyration and the forward scattering intensity, *I*(0), were evaluated with the program PRIMUS.^45^ Molecular masses were calculated from the *I*(0) values, normalized to the *I*(0) for bovine serum albumin. The distance distribution function *P*(*r*) was generated with the program GNOM.^19^ Particle shapes were restored from the experimental scattering profiles using *ab initio* modeling with DAMMIF^20^ imposing 6-fold symmetry. Ten independent DAMMIF runs were performed for each protein; these generated very similar but not identical shapes in each case. An averaged filtered shape was generated using DAMAVER and DAMFILT.^46^

### PONDR analysis

Potentially disordered regions of VcHfq and EcHfq were identified by the PONDR VL-XT algorithm.^23–25^ Access to PONDR was provided by Molecular Kinetics† (Indianapolis, IN) under license from the Washington State University Research Foundation (VL-XT is copyright 1999 by the Washington State University Research Foundation, all rights reserved; PONDR is copyright 2004 by Molecular Kinetics, all rights reserved).

### CD spectroscopy

CD data were collected at 20 °C on an Applied Photophysics π⁎-180 spectrometer for VcHfq and EcHfq in 1 mM Tris, pH 8, and 100 mM KF. The protein concentrations were 0.157 mg/ml, 0.475 mg/ml, and 1.475 mg/ml for VcHfq for cuvettes with 0.2-mm, 0.5- mm, and 1- mm path lengths, respectively. For EcHfq, the concentrations were 0.417 mg/ml, 0.833 mg/ml, 0.051 mg/ml, 0.026 mg/ml, and 0.101 mg/ml for cuvettes with 0.1- mm, 0.2- mm, 1- mm, 4- mm, and 10- mm path lengths, respectively. Data were collected to span wavelengths from 180 nm to 360 nm in 1- nm steps. Data from four or six scans were averaged, baseline subtracted, smoothed using the Savitzky–Golay routine, and calibrated against camphor sulfonic acid. The spectra were converted into molar ellipticity units (deg cm^2^ dmol^− 1^), and the secondary-structure content was extracted by iterative fitting to basis sets from Compton and Johnson^47^ [basis sets for helix, irregular (core), β-turn, antiparallel β sheets and parallel β-sheets] and Brahms and Brahms^48^ [basis set for irregular (random) protein tails].

### SDS-PAGE stability assay

One microgram  of each Hfq protein was added to 10 μl SDS-PAGE loading buffer (50 mM Tris, pH 6.8, 6% w/v glycerol, 0.01% w/v bromophenol blue, and 1% w/v SDS) and heated at 95 °C for 0, 10, 20, 30, 45, 60, 75, 90, 120, 180, or 300 s. Samples were analyzed by 10% SDS-PAGE (gels contained 0.1% SDS) stained with SimplyBlue SafeStain (Invitrogen). Gels were quantified using Multi Gauge software v2.2 (Fujifilm), and the half-life of the hexamer in 1% SDS at 95 °C was determined, from experiments repeated in triplicate, using GraFit v5.0.11 (Erithacus Software Limited).

### Electrophoretic mobility shift assay

DNA template encoding Qrr1 sRNA preceded by the T7 promoter sequence was generated by gene synthesis from overlapping primers^42^ using Qrr1 primers (Table S2). Three guanine nucleotides were added to the 5′ end of the Qrr sequence to ensure efficient transcription by T7 RNA polymerase. ^32^P-labelled Qrr1 sRNA was prepared by *in vitro* transcription from this DNA template using the MaxiScript T7 Kit (Applied Biosystems) and [α-^32^P]UTP (PerkinElmer). Unincorporated [α-^32^P]UTP was removed with an illustra MicroSpin G-25 column (GE Healthcare). RNA was heated to 80 °C for 10 min and allowed to cool to room temperature before use. Ten-microliter  reactions were assembled containing 5 nM ^32^P-labelled Qrr1, the indicated concentration of Hfq (hexamer), 20 mM Tris, pH 8, 50 mM KCl, 50 mM NaCl, 0.5 mM EDTA, and 10% glycerol. Reactions were incubated at room temperature for 30 min and analyzed by 6% PAGE at room temperature using TBE buffer. Gels were visualized using a FLA-5000 Fluoro Image Analyzer (Fujifilm) and quantified using Multi Gauge software v2.2. *K*_d_ values were determined, from experiments repeated six times, using GraFit v5.0.11.

## Figures and Tables

**Table 1:** Secondary-structure content of VcHfq and EcHfq determined by CD spectroscopy

Protein	Secondary-structure content, number of amino acids (%)			
	α-Helix	β-Sheet	β-Turn	Other/Irregular
VcHfq	13–14 (15)	41–42 (45)	9 (10)	27 (30)
EcHfq	15 (15)	41 (40)	10 (10)	36 (35)

**Table 2:** Half-life of Hfq hexamers in 1% SDS at 95 °C

Protein	VcHfq	VcHfq72	VcNTDEcCTR	EcHfq	EcHfq72	EcNTDVcCTR
Half-life of hexamer in 1% SDS at 95 °C (s)	33.2 ± 1.3	32.1 ± 1.9	39.2 ± 1.4	49.6 ± 0.5	41.5 ± 2.4	43.0 ± 1.8

**Table 3:** Hfq binding to Qrr1 sRNA

Protein	VcHfq	VcHfq72	VcNTDEcCTR	EcHfq	EcHfq72	EcNTDVcCTR
*K*_d_ for 1:1 complex (nM)	31.1 ± 1.2	44.4 ± 3.0	18.0 ± 0.9	18.7 ± 1.3	40.8 ± 2.3	28.0 ± 1.0
*K*_d_ for higher-order complex(es) (nM)	204 ± 17	40.2 ± 2.1	151 ± 15	416 ± 67	35.0 ± 1.8	184 ± 15

## Footnotes

† www.molecularkinetics.com; E-mail: main@molecularkinetics.com.
