# Supplementary material for: Characterization of Vibrio cholerae Hfq Provides Novel Insights into the Role of the Hfq C-Terminal Region
Source: J Mol Biol. 2012 Jun 29;420(1-2):56–69. doi: 10.1016/j.jmb.2012.03.028 (PMC3477312; doi:10.1016/j.jmb.2012.03.028)
Supplement: Supplementary file 1 — Supplementary materials [file mmc1.pdf]

## SUPPLEMENTARY DATA

**Figure S1 Taxonomic Families of Gammaproteobacteria Can Be Recovered Using Hfq DNA Sequences**

54 non-redundant (<95% identical) *hfq* DNA and corresponding protein sequences of representative species of eight gammaproteobacteria families were analysed (Table S1). *hfq* DNA sequences were retrieved from the NCBI nucleotide database using BlastN and the sequence of *E. coli hfq* as the query. Gammaproteobacteria are classified into 13 orders in accordance to their distribution in 16S rRNA gene trees[1, 2]. However, phylogenies computed using multiple concatenated proteins, an approach that is considered to be more robust, were unable to recover some of these taxons[3, 4]; for this reason, we restricted our analysis to taxonomic families that have not shown conflicting phylogenies (Table S1). Phylogenies were calculated for both amino acid and DNA sequences. The best model for amino acid replacement was selected using Prottest 1.4[5] and was Dayhoff with gamma-distributed rate variation. The best model for DNA substitution was selected using MrAIC 1.4.3[6] and was symmetrical with gamma distribution of rate variation and a proportion of invariable sites. Phylogenetic analyses of the DNA sequences corresponding to Hfq where performed both by maximum likelihood and Bayesian approaches. Maximum likelihood trees were estimated with PhyML 2.4.4[7] and a Bayesian analysis was performed with MrBayes[8]. Bootstrap replicates and consensus trees were calculated with the Phylip suite[9]. Trees were visualized and prepared for publication with Dendroscope[10].

(a) Maximum-likelihood consensus phylogenetic tree computed using PhyML 2.4.4[7] with 1000 bootstrap replicates and a 50% majority-rule. Numbers indicate percentage of support, only

those equal to or more than 50% are shown. Sequences used in the analysis are listed in Table S1.

(b) Consensus phylogenetic tree computed using MrBayes[8] with default parameters for five million generations sampling every 200 generations. The first 6250 trees were discarded before computing the majority-rule consensus tree. Numbers indicate credibility (posterior probability) values. Sequences used in the analysis are listed in Table S1.

**Figure S2 Multiple Sequence Alignment of Representative Members of the Gammaproteobacteria Families.**

Only non-redundant sequences (<95% amino acid identity) are shown. Positions are coloured according to the Clustal colour scheme: glycines are orange and prolines are yellow; other positions are coloured according to conservation of chemical properties: hydrophobic in blue, aromatic in cyan, polar negative in purple, polar positive in red and polar neutral in green.

**Figure S3 Analytical Ultracentrifugation (AUC) of VcHfq and EcHfq**

AUC was carried out in a Beckman Optima XL-A analytical ultracentrifuge using an AnTi-50 eight-hole rotor (Beckman Coulter). Sedimentation velocity experiments used double-sector cells with a 12 mm path length with 400 µl of VcHfq at 3.29 mg/ml or EcHfq at 4.13 mg/ml in 10 mM Tris pH 8, 50 mM NaCl and 50 mM KCl buffer loaded into the sample channel and 425 µl of buffer loaded into the buffer channel. The rotor was accelerated to 30,000 rpm at 10ºC. Radial absorbance scans were collected every 10 minutes at 280 nm. The differential sedimentation coefficient distribution (c(S)) and the molar mass distribution (c(M)) were calculated in SEDFIT[2] using a partial specific volume of 0.73 mg/ml. Sedimentation

equilibrium experiments used six-channel cells with a 12 mm path length. 90 µl of VcHfq at 3.29 mg/ml or EcHfq at 4.13 mg/ml in 10 mM Tris pH 8, 50 mM NaCl and 50 mM KCl buffer were loaded into the sample channels and 110 µl of buffer were loaded into the buffer channels. The rotor was accelerated at 20,000 rpm at 10ºC for 24 hours. Scans of absorbance versus radial displacement were measured at 280 nm after 18, 21 and 24 hours. Data were analysed in Origin 6.0 (Microcal Software Inc., developed by Beckman Coulter) and molecular masses were calculated using a partial specific volume of 0.73 ml/g.

(a) Sedimentation velocity. (i) Fits of the data for VcHfq and EcHfq. For clarity, only every fourth scan is shown. The residual error between the fit and the experimental data is shown below the fit for each protein. (ii) The associated c(S) distribution plot for VcHfq (blue) and EcHfq (magenta). The sedimentation coefficient is indicated above the respective peak. (iii) The associated c(M) distribution plot for VcHfq (blue) and EcHfq (magenta). The molecular mass is indicated above the respective peak. The theoretical molecular mass is 60,981.6 Da for VcHfq and 66,210.6 Da for EcHfq.

(b) Sedimentation equilibrium. Example fits (lower panels) are shown for data scanned at 280 nm after 24 hours for VcHfq and EcHfq. The residual error between the fit and the experimental data is shown for each protein in the upper panels. The theoretical molecular mass is 60,981.6 Da for VcHfq and 66,210.6 Da for EcHfq.

**Figure S4 Non-denaturing MS Reveals that the VcHfq Hexamer is Less Stable than the EcHfq Hexamer**

Non-denaturing MS was performed using 10 µM solutions of VcHfq and EcHfq in aqueous ammonium acetate buffer pH 7 (for MS mode 83 mM, for MS/MS mode 250 mM).

Samples were introduced using nano-electrospray ionization (ESI) with a TriVersa Nanomate inlet system (Advion) and a Synapt T-wave Ion Mobility Mass Spectrometer (IM-MS; Waters) in positive ion mode. Experimental parameters were chosen to preserve intact non-covalent interactions during transfer of ions into the mass spectrometer[11]. In MS/MS mode the 16+ peaks of both protein hexamers were chosen for collision-induced dissociation (CID) and the release of the monomer was monitored. Key settings were backing: 3 mbar and source: 1.6 x $10^{-3}$ mbar; in MS mode: sampling cone: 75 V, extraction cone: 1 V, trap and transfer collision energy: 9 and 6 V respectively, bias: 17.5 V; in MS/MS mode: sampling cone: 50 V, extraction cone: 0 V, trap collision energy: variable, transfer collision energy: 3 V, bias: 15 V. Data were smoothed using a window with ± 50 channels using MassLynx software, 4.0.

(a) Non-denaturing MS of VcHfq (left) and EcHfq (right) under identical conditions. The peak series corresponding to the intact hexamer are labelled with H in both spectra and those corresponding to monomer are labeled with M. The VcHfq spectra shows a series of peaks which correspond to the 14+ to 18+ charge states of hexameric protein next to a much smaller series centred on the 8+ charge state corresponding to the monomer (theoretical mass: 10,163.6 Da; experimental mass: 10,165.9 Da). Similarly, the EcHfq shows a peak series corresponding to the 15+ to 18+ charge states of the hexamer and a series centred on the 8+ charge state of the monomeric species (theoretical mass: 11,035.1 Da; experimental mass: 11,037.0 Da). Additional species, marked with an * are most likely due to minor impurities co-purifying with the VcHfq/EcHfq hexamers. The peak series labeled with # in each spectrum is attributed to a very small amount of dodecamer, which has been observed previously for *E. coli* Hfq[12, 13]. The 16+ charge states, which are highlighted in bold, were chosen for fragmentation by collision induced dissociation (CID) prior to the ion mobility cell (b).

(b). Fragment MS/MS spectra for VcHfq (left column) and EcHfq (right column) for three different trap collision energies: 45 V (top panels), 55 V (middle panels) and 120 V (bottom panels).

## REFERENCES


1. Kersters, K., de Vos, P., Gillis, M., Swings, J., Vandamme, P. & Stackebrand, E. (2006). Introduction to the Proteobacteria. In The Prokaryotes: A Handbook on the Biology of Bacteria (Dworkin, M., Falkow, S., Rosenberg, E., Schleifer, K. H., Stackebrandt, E., ed.). 3-37, Springer, New York, USA.

2. Garrity, G. M., Bell, J. A., Lilbum, T., Brenner, D. J., Krieg, N. R. & Staley, J. T. (2005). The Revised Road Map to the Manual. In Bergey's Manual of Systematic Bacteriology. 159-187, Springer, NewYork, USA.

3. Williams, K. P., Gillespie, J. J., Sobral, B. W., Nordberg, E. K., Snyder, E. E., Shallom, J. M. & Dickerman, A. W. Phylogeny of gammaproteobacteria. *J. Bacteriol.* **192**, 2305-2314.

4. Gao, B., Mohan, R. & Gupta, R. S. (2009). Phylogenomics and protein signatures elucidating the evolutionary relationships among the Gammaproteobacteria. *Int. J. Syst. Evol. Microbiol.* **59**, 234-247.

5. Abascal, F., Zardoya, R. & Posada, D. (2005). ProtTest: selection of best-fit models of protein evolution. *Bioinformatics.* **21**, 2104-2105.

6. Nylander, J. A. A. (2004). MrAIC [1.4.3]. *Evolutionary Biology Centre, Uppsala University*.

7. Guindon, S., Delsuc, F., Dufayard, J. F. & Gascuel, O. (2009). Estimating maximum likelihood phylogenies with PhyML. *Methods Mol. Biol.* **537**, 113-137.

8. Ronquist, F. & Huelsenbeck, J. P. (2003). MrBayes 3: Bayesian phylogenetic inference under mixed models. *Bioinformatics.* **19**, 1572-1574.

9. Felsenstein, J. (2005). PHYLIP (Phylogeny Inference Package) [3.6]. *Department of Genome Sciences, University of Washington, Seattle*.

10. Huson, D. H., Richter, D. C., Rausch, C., Dezulian, T., Franz, M. & Rupp, R. (2007). Dendroscope: An interactive viewer for large phylogenetic trees. *BMC Bioinformatics* **8**, 460.

11. Sobott, F., McCammon, M. G., Hernández, H. & Robinson, C. V. (2005). The flight of macromolecular complexes in a mass spectrometer. *Phil. Trans. R. Soc. A* **363**, 379-391.

12. de Haseth, P. L. & Uhlenbeck, O. C. (1980). Interaction of *Escherichia coli* host factor protein with Q beta ribonucleic acid. *Biochemistry* **19**, 6146-6151.

13. de Haseth, P. L. & Uhlenbeck, O. C. (1980). Interaction of *Escherichia coli* host factor protein with oligoriboadenylates. *Biochemistry* **19**, 6138-6146.

**Table S1 Hfq Sequences for Phylogenetic Analysis and Multiple Sequence Alignmnet**

| Taxonomic family/Tree group | Species | NCBI accession code | Position of gene in accession code sequence |
|---|---|---|---|
| Ectothiorhodospiraceae | *Alkalilimnicola ehrlichii* MLHE-1 | 114225560 | 622297-622566 |
| Ectothiorhodospiraceae | *Halorhodospirahalophila* SL1 | 121588215 | 748883-749155 |
| Ectothiorhodospiraceae | *Thioalkalivibrio* K90 mix | 288942818 | 517278-517532 |
| Enterobateriaceae | *Cronobacter turicensis* z3032 | 323575285 | 3838103-3838411 |
| Enterobateriaceae | *Dickeya dadantii* 3937 | 307128764 | 4253799-4254098 |
| Enterobateriaceae | *Enterobacter cloacae* subsp *cloaeae* ATCC | 295054830 | 563135-563446 |
| Enterobateriaceae | *Erwinia billingiae* st Eb661 | 299060424 | 540494-540802 |
| Enterobateriaceae | *Erwinia tasmaniensis* | 188027207 | 3307510-3307818 |
| Enterobateriaceae | *Escherichia coli* | 332341332 | 4988603-4988911 |
| Enterobateriaceae | *Klebsiella variicola* At22 | 288887617 | 4972955-4973263 |
| Enterobateriaceae | *Rahnella* Y9602 | 321165934 | 459891-460199 |
| Enterobateriaceae | *Serratia plymuthica* G3 | 193888381 | - |
| Enterobateriaceae | *Sodalis glossinidius* str *morsitans* | 84778498 | 623805-624110 |
| Enterobateriaceae | *Yersinia pestis* KIM D27 | 270335045 | 2634180-2634485 |
| Moraxellaceae | *Acinetobacter baumannii* TCDC-AB0715 | 323516058 | 2650109-2650615 |
| Moraxellaceae | *Acinetobacter calcoaceticus* PHEA-2 | 325121063 | 1713026-1713550 |
| Moraxellaceae | *Psychrobacter cryohalolentis* K5 | 92392509 | 1836200-1836751 |
| Pasteurellaceae | *Actinobacillus pleuropneumoniae* str AP76 | 189914400 | 2253347-2253625 |
| Pasteurellaceae | *Actinobacillus succinogenes* 130Z | 150839411 | 1065965-1066264 |
| Pasteurellaceae | *Aggregatibacter actinomycetemcomitans* D11S-1 | 261412053 | 877097-877393 |
| Pasteurellaceae | *Aggregatibacter aphrophilus* NJ8700 | 247533203 | 610066-610368 |
| Pasteurellaceae | *Haemophilus ducreyi* 35000HP | 33149228 | 582457-582762 |
| Pasteurellaceae | *Haemophilus influenzae* F3031 | 317431924 | 1843745-1844020 |
| Pasteurellaceae | *Haemophilus parainfluenzae* T3T1 | 301154649 | 1077751-1078035 |
| Pasteurellaceae | *Haemophilus parasuis* SH0165 | 219690483 | 1194908-1195189 |
| Pasteurellaceae | *Haemophilus somnus* 2336 | 168825335 | 1235543-1235833 |
| Pasteurellaceae | *Mannheimia succiniciproducens* MBEL55E | 52306107 | 1508974-1509264 |
| Pasteurellaceae | *Pasteurella multocida multocida* str Pm70 | 13400023 | 1064798-1065088 |
| Pseudomonadaceae | *Azotobacter vinelandii* DJ | 226717097 | 713089-713343 |
| Pseudomonadaceae | *Pseudomonas aeruginosa* LESB58 | 218768969 | 5880732-5880980 |
| Pseudomonadaceae | *Pseudomonas entomophila* str L48 | 95101722 | 5247772-5248032. |
| Pseudomonadaceae | *Pseudomonas mendocina* ymp | 145573243 | 702481-702741 |
| Pseudomonadaceae | *Pseudomonas putida* BIRD | 313496345 | 5223576-5223836-1 |
| Pseudomonadaceae | *Pseudomonas stutzeri* A1501 | 145568602 | 3963634-3963888 |
| Shewanellaceae | *Shewanella amazonensis* SB2B | 119765642 | 3599691-3599966 |
| Shewanellaceae | *Shewanella loihica* PV-4 | 126636230 | 642172-642450 |
| Shewanellaceae | *Shewanella pealeana* ATCC | 157844830 | 4323638-4323916 |
| Shewanellaceae | *Shewanella piezotolerans* WP3 | 212554395 | 790124-790402 |
| Vibrionaceae | *Aliivibrio salmonicida* LFI1238 | 208007585 | 3007118-3007384 |
| Vibrionaceae | *Listonella anguillarum* | 167987245 | 375-641 |
| Vibrionaceae | *Photobacterium profundum* SS9 | 46914592 | 314528-314785 |
| Vibrionaceae | *Vibrio cholerae* MJ-1236 | 229368777 | 269828-270091 |
| Vibrionaceae | *Vibrio fischeri* ES114 | 171902228 | 2610406-2610672 |
| Vibrionaceae | *Vibrio furnissii* NCTC 11218 | 315178329 | 11716-11979 |
| Vibrionaceae | *Vibrio parahaemolyticus* RIMD 2210633 | 47118310 | 2980906-2981169 |
| Vibrionaceae | *Vibrio splendidus* LGP32 | 223587976 | 263140-263406 |
| Vibrionaceae | *Vibrio vulnificus* CMCP6 | 319998996 | 1280011-1280271 |
| Xanthomonadaeae | *Pseudoxanthomonas suwonensis* 11 | 317464132 | 893565-893840-1 |
| Xanthomonadaeae | *Stenotrophomonas maltophilia* R551 | 194346582 | 1654093-1654368-3 |
| Xanthomonadaeae | *Xanthomonas albilineans* str GPE PC73 | 283472039 | 1365965-1366240 |
| Xanthomonadaeae | *Xanthomonas campestris* str ATCC | 21166373 | 1991534-1991812 |
| Xanthomonadaeae | *Xanthomonas oryzae* MAFF 31018 | 84365597 | 3149285-3149563 |
| Xanthomonadaeae | *Xylella fastidiosa* M12 | 167964044 | 87835-88113 |

**Table S2 Primers Used to Construct Hfq Expression Plasmids and DNA Template for *in vitro* Transcription of Qrr1**

| Primer Name | Primer Sequence (5' to 3') |
|---|---|
| VcHfqf | tac gta cat atg gct aag ggg caa tct c |
| VcHfqr | tac gta ctc gag tca caa ctc ttc aga ctt ctc tgc |
| VcHfq72f | cct gct cgt cca gtt agc cac cac agc taa cat atg ggc gac cgc cca gca tcg gat c |
| VcHfq72r | gat ccg atg ctg ggc ggt cgc cca tat gtt agc tgt ggt ggc taa ctg gac gag cag g |
| VcNTDEcCTRS2 | cct gct cgt cca gtt agc cac cac agc aac aac g |
| VcNTDEcCTRS1 | gcc acc aca gca aca acg ccg gtg gcg gta cca gca gta act acc atc atg gta gca gcg |
| VcNTDEcCTRAS1 | cgg ttt ctt cgc tgt cct gtt gcg cgg aag tat tct gcg cgc tgc tac cat gat ggt agt |
| VcNTDEcCTRAS2 | gat ccg atg ctg ggc ggt cgc cca tat gtt att cgg ttt ctt cgc tgt cct gt |
| | |
| EcHfqf | tac gta cat atg gct aag ggg caa tct |
| EcHfqr | tac gta ctc gag tca tta ttc ggt ttc ttc |
| EcHfq72f | ccc gtc tcg ccc ggt ttc tca tca cag t taa cat atg aac aac gcc ggt ggc ggt acc agc ag |
| EcHfq72r | ctg ctg gta ccg cca ccg gcg ttg ttc ata tgt taa ctg tga tga gaa acc ggg cga gac ggg |
| EcNTDVcCTRS2 | ccc gtc tcg ccc ggt ttc tca tca |
| EcNTDVcCTRS1 | ctc gcc cgg ttt ctc atc aca gtg gcg acc gcc cag cat cgg atc gtc cag cag a |
| EcNTDVcCTRAS1 | cca ccg gcg ttg ttc ata tgt tac tct tca gac ttc tct gct gga cga tcc gat g |
| EcNTDVcCTRAS2 | gat ccg atg ctg ggc ggt cgc cct gct ggt acc gcc acc ggc gtt gtt cat atg |
| | |
| Qrr1S2 | taa tac gac tca cta tag ggt gac ccg caa ggg tca c |
| Qrr1S1 | gtg acc cgc aag ggt cac cta gcc aac tga cgt tgt tag tga ata atc |
| Qrr1AS1 | gaa tga gtc tat tgg ctg tta ttt gtg aac att gat tat tca cta aca acg tca gtt g |
| Qrr1AS2 | aaa aaa ata gcc aat aga atg agt cta ttg gct gtt att tgt gaa cgc |

# Figure S1

a

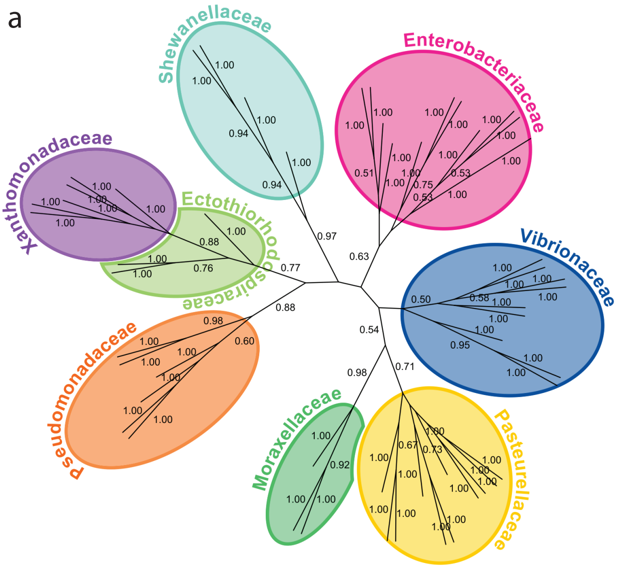
Shewanellaceae
Enterobacteriaceae
Xanthomonadaceae
Ectothiorhodospiraceae
Vibrionaceae
Pseudomonadaceae
Moraxellaceae
Pasteurellaceae

b

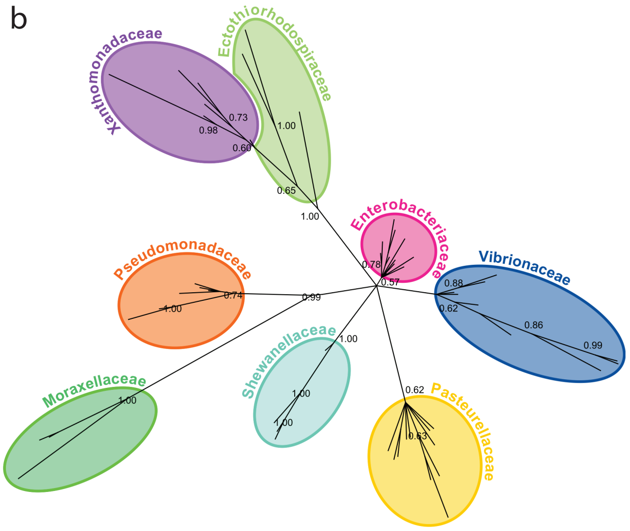
Xanthomonadaceae
Ectothiorhodospiraceae
Enterobacteriaceae
Pseudomonadaceae
Vibrionaceae
Moraxellaceae
Shewanellaceae
Pasteurellaceae

# Figure S2

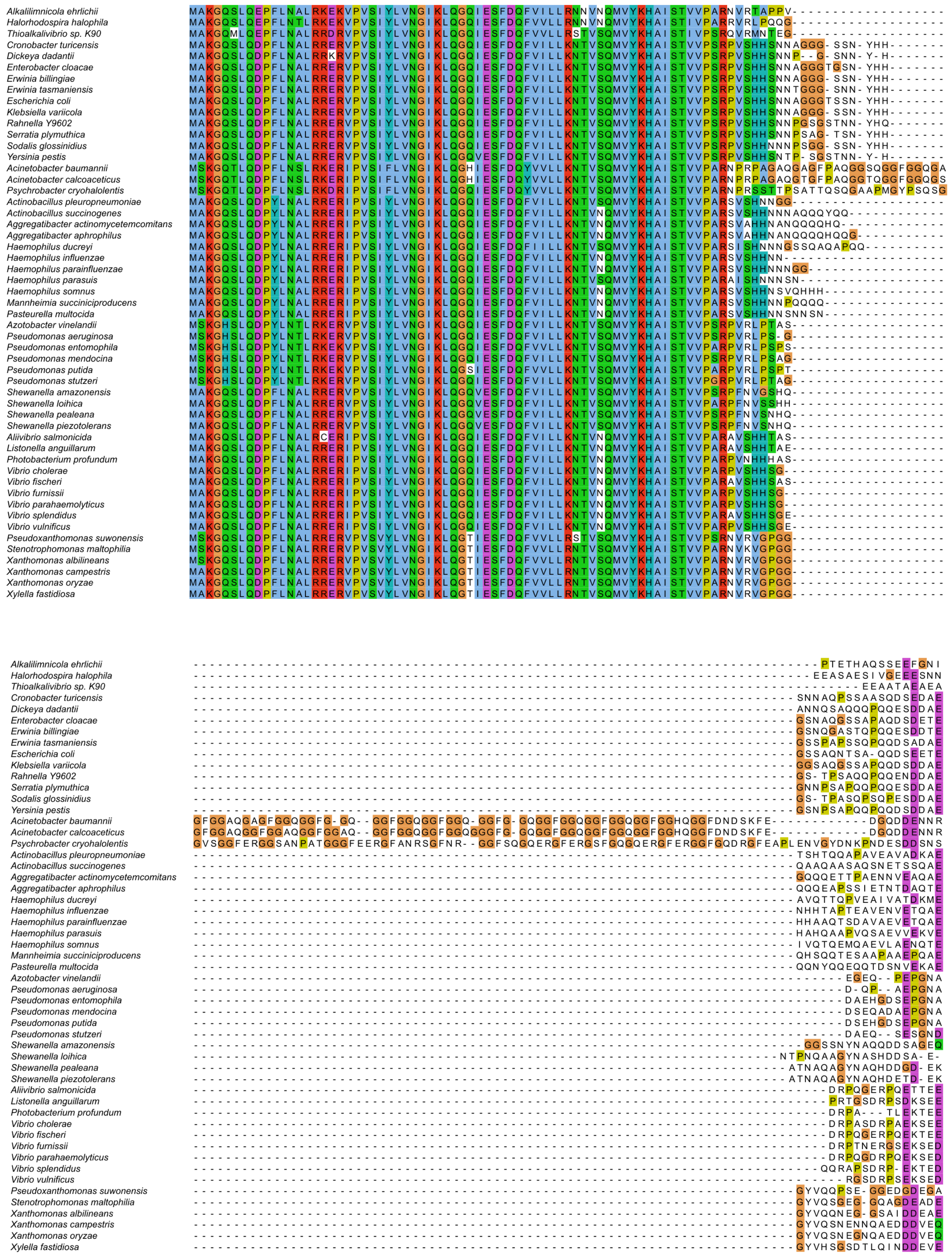
Alkalilimnicola ehrlichii
Halorhodospira halophila
Thioalkalivibrio sp. K90
Cronobacter turicensis
Dickeya dadantii
Enterobacter cloacae
Erwinia billingiae
Erwinia tasmaniensis
Escherichia coli
Klebsiella variicola
Rahnella Y9602
Serratia plymuthica
Sodalis glossinidius
Yersinia pestis
Acinetobacter baumannii
Acinetobacter calcoaceticus
Psychrobacter cryohalolentis
Actinobacillus pleuropneumoniae
Actinobacillus succinogenes
Aggregatibacter actinomycetemcomitans
Aggregatibacter aphrophilus
Haemophilus ducreyi
Haemophilus influenzae
Haemophilus parainfluenzae
Haemophilus parasuis
Haemophilus somnus
Mannheimia succiniciproducens
Pasteurella multocida
Azotobacter vinelandii
Pseudomonas aeruginosa
Pseudomonas entomophila
Pseudomonas mendocina
Pseudomonas putida
Pseudomonas stutzeri
Shewanella amazonensis
Shewanella loihica
Shewanella pealeana
Shewanella piezotolerans
Aliivibrio salmonicida
Listonella anguillarum
Photobacterium profundum
Vibrio cholerae
Vibrio fischeri
Vibrio furnissii
Vibrio parahaemolyticus
Vibrio splendidus
Vibrio vulnificus
Pseudoxanthomonas suwonensis
Stenotrophomonas maltophilia
Xanthomonas albilineans
Xanthomonas campestris
Xanthomonas oryzae
Xylella fastidiosa

Figure S3

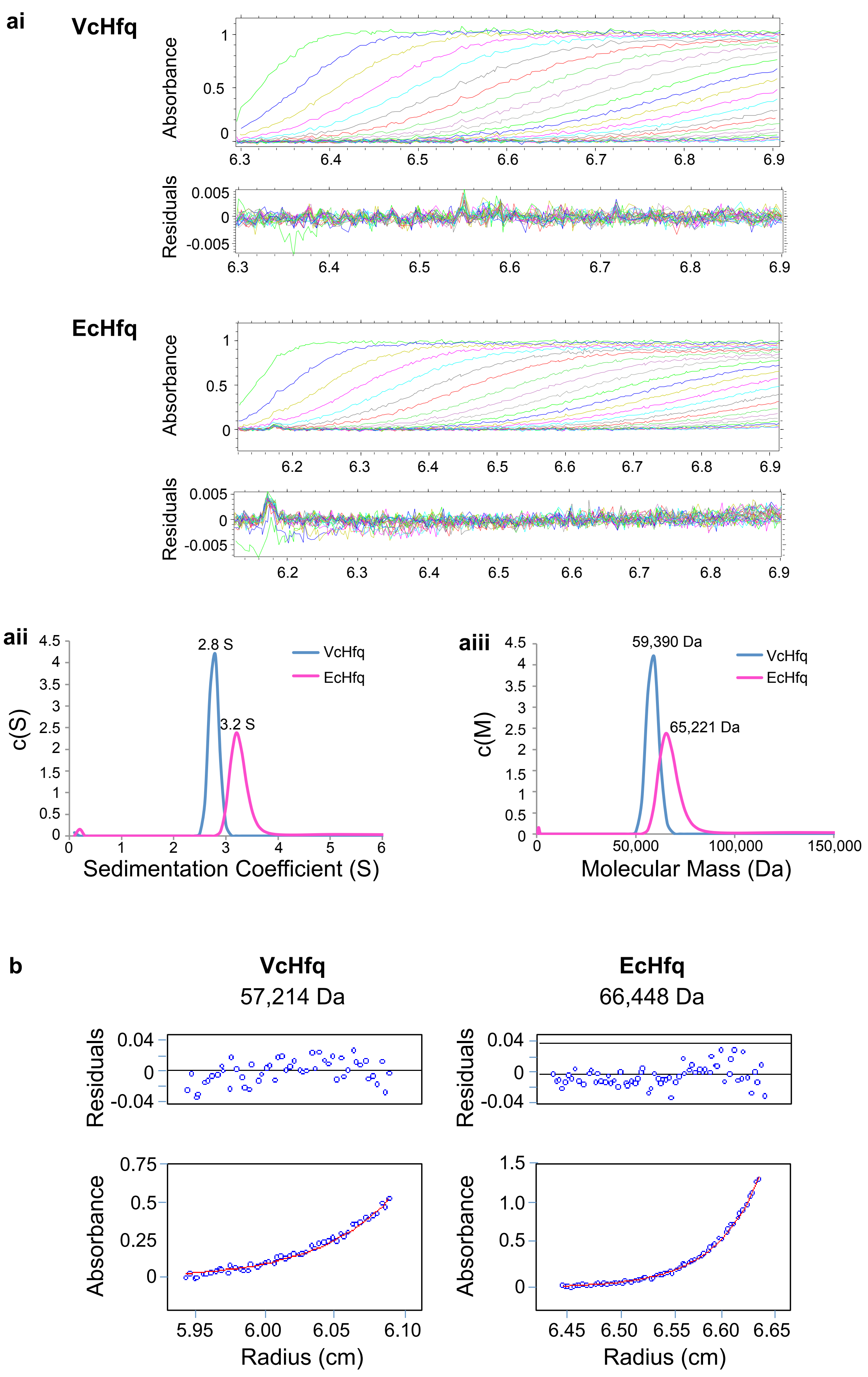

ai
VcHfq
Absorbance
Residuals
EcHfq
aii
c(S)
2.8 S
3.2 S
VcHfq
EcHfq
Sedimentation Coefficient (S)
aiii
c(M)
59,390 Da
65,221 Da
Molecular Mass (Da)
b
VcHfq
57,214 Da
EcHfq
66,448 Da
Radius (cm)

Figure S4

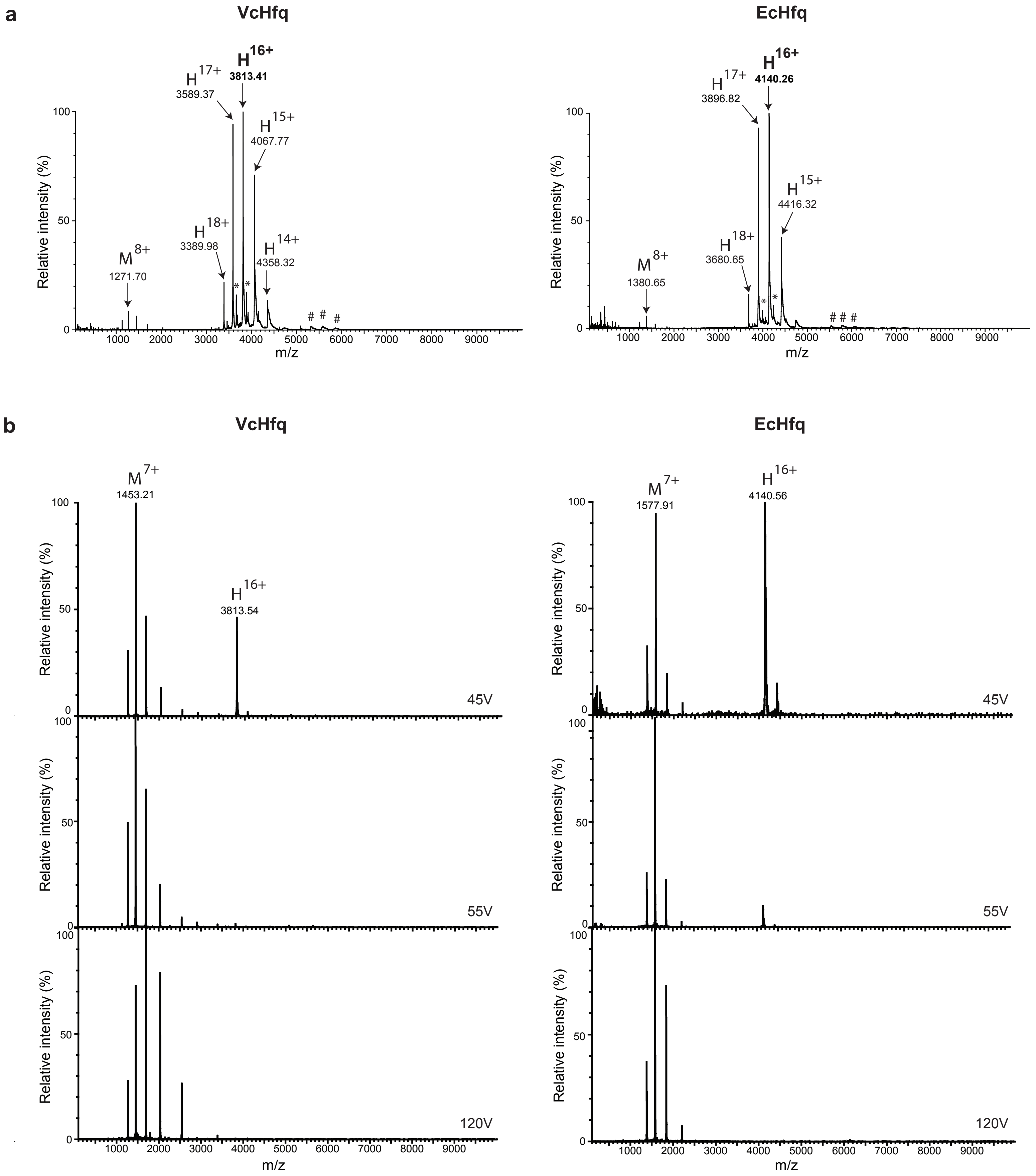

a
VcHfq
EcHfq
H16+
3813.41
H17+
3589.37
H15+
4067.77
H18+
3389.98
H14+
4358.32
M8+
1271.70
H16+
4140.26
H17+
3896.82
H15+
4416.32
H18+
3680.65
M8+
1380.65
Relative intensity (%)
m/z
b
VcHfq
EcHfq
M7+
1453.21
H16+
3813.54
M7+
1577.91
H16+
4140.56
45V
55V
120V
Relative intensity (%)
m/z
